# MiR-573 suppresses cell proliferation, migration and invasion via regulation of E2F3 in pancreatic cancer

**DOI:** 10.7150/jca.51147

**Published:** 2021-03-19

**Authors:** Zhou Pengcheng, Gao Peng, Fan Haowen, Lin Xida, Lu Yuhua, Wang Yao, Zhu Mingyan, Fan Xiangjun, Wang zhiwei, Zhang Yewei, Wang Lei

**Affiliations:** 1 Medical school of Southeast University, Nanjing, Jiangsu, China.; 2 Affiliated Hospital of Nantong University, Nantong, Jiangsu, China.; 3 Nantong Traditional Chinese Medicine Hospital, Nantong, Jiangsu, China.; 4 Nantong University, Nantong, Jiangsu, China.; 5 Zhongda Hospital affiliated to Southeast University, Nanjing, Jiangsu, China.

**Keywords:** pancreatic cancer, miR-573, E2F3, proliferation, invasion

## Abstract

**Background:** Pancreatic cancer is among the most lethal malignancies worldwide. In this study, we aimed to determine whether miR-573 could suppress pancreatic cancer cell proliferation, migration, and invasion by targeting E2F3.

**Materials and Methods:** MiR-573 expression in pancreatic cancer tissues and cell lines was measured using real-time PCR. Target genes of miR-573 were screened using bioinformatics tools and confirmed using dual-luciferase reporter assay and real-time PCR. Pancreatic cancer cells were transfected using an miR-573 mimic or siRNA E2F3. Furthermore, cell proliferation, migration, and invasion were assessed using CCK-8, Edu staining, colony-forming assay, wound healing assay, and transwell assay *in vitro*. The *in vivo* effects of miR-573 were verified using tumor xenografts. Differential expression and prognostic analyses of miR-573 and E2F3 were visualized using the Kaplan‑Meier plotter and GEPIA.

**Results:** We found that the expression of miR-573 was significantly reduced in pancreatic cancer tissues and cell lines. Overexpression of miR-573 obviously suppressed the proliferation, migration, and invasion of pancreatic cancer cells. The Dual-luciferase assay showed that miR-573 could specifically target E2F3. Furthermore, E2F3 was up-regulated in pancreatic cancer tissues and cell lines and E2F3 down-regulation inhibited the proliferation, migration, and invasion of pancreatic cancer cells. The ectopic expression of miR-573 inhibited xenograft tumor growth *in vivo*. Results from the Kaplan-Meier analysis and GEPIA showed that patients with a high level of miR-573 had a significantly reduced risk of death while those with a high level of E2F3 displayed significant correlation with the tumor stage and suffered worse prognosis.

**Conclusions:** MiR-573 could suppress the proliferation, migration, and invasion of pancreatic cancer cells by targeting E2F3, thereby establishing miR-573 as a novel regulator of E2F3 and indicating its critical role in tumorigenesis, especially in pancreatic cancer.

## Introduction

Pancreatic cancer is considered to be one of the most lethal malignancies worldwide with a five-year survival lower than 5% 1. It is the fourth leading cause of cancer-related deaths and is expected to become the second by 2030 2]. At present, the primary treatment for pancreatic cancer is surgery followed by chemotherapy and palliative care. However, the majority of patients are diagnosed at an advanced stage, and less than 20% of these patients are candidates for surgical resection. Even after R0 resection, most patients eventually suffer from recurrence. The poor prognosis for pancreatic cancer can be attributed to the difficulty in early diagnosis, local recurrence, rapid metastasis, and resistance to conventional chemotherapy. Thus, the identification of new biomarkers, treatments, and therapeutic targets for pancreatic cancer is essential.

MicroRNAs (miRNAs) are highly conserved, small noncoding RNAs with a length of 18-24 nucleotides 3. They bind to the 3'-UTR of target mRNAs, which causes binding RNA degradation or inhibition of its translation, thus silencing mRNA targets 4. Emerging evidence shows that miRNAs play a crucial role in various physiological and pathological processes, such as cell growth, proliferation, differentiation, and apoptosis. Aberrant expression of miRNAs is observed in different types of cancers, including pancreatic cancer, and is closely related to cancer progression, functioning as either tumor suppressors or oncogenes. In pancreatic cancer, miRNAs have been identified to be abnormally expressed; thus, may serve as potential therapeutic targets 5. Recently, it was found that miR-573 regulates cell proliferation and apoptosis by targeting Bax in nucleus pulposus cells 6. Besides, studies have demonstrated that miR-573 acts as a tumor suppressor in the development of several cancers, including lung cancer 7, cervical cancer 8, prostate cancer 9, and hepatocellular carcinoma 10. Nevertheless, the specific biological functions of miR-573 in pancreatic cancer have not been investigated yet.

E2F transcription factor 3 (E2F3) is a transcription factor that influences cell cycle regulation and apoptosis and controls various biological and physiological processes, such as DNA synthesis and repair, and centrosome duplication 11. It is commonly dysregulated in cancers of the lung 12, ovary 13, bladder 14, and stomach 15 and plays an oncogenic role. Nevertheless, the biological relevance of miR‑573/E2F3 in pancreatic cancer remains unclear.

In the current study, we analyzed the expression of miR-573 and E2F3 in pancreatic cancer tissues and determined their role in the progression of pancreatic cancer. We identified that E2F3 was a direct downstream target of miR-573. Collectively, these findings suggest that targeting miR-573/E2F3 signaling may be a potential strategy in the treatment of pancreatic cancer.

## Materials and methods

### Clinical tissue collection

From July 2017 to July 2019, 41 pancreatic cancer specimens and adjacent normal tissues were obtained from patients who underwent surgery at Affiliated Hospital of Nantong University (Jiangsu, China). The inclusion criteria were as follows: Patients aged over 18 years old, who were diagnosed as PDAC based on histopathological evaluation, and the clinical data of the patients were complete. In contrast, the exclusion criteria were as follows: Patients associated with other malignant tumors or a history of malignancy, patients with liver and kidney disease, patients with infectious disease, patients with previous treatment history (radiotherapy, chemotherapy, radiofrequency ablation, hepatic artery embolization chemotherapy, anhydrous alcohol injection or antibiotic treatment) before this study. Tissues were immediately frozen in liquid nitrogen after leaving the bodies and stored in a freezer at -80 °C until use. All resected tissues were postoperatively diagnosed with pancreatic cancer. All patients gave written informed consent. The Ethics Committee of the Affiliated Hospital of Nantong University approved this study. MicroRNA array data was obtained from the GEO dataset GES43796. No ethical issues were involved, because the array data were obtained by using the GEO database.

### Cell culture

Three human pancreatic cancer cell lines, namely, PANC‑1, CFPAC-1, and MIAPaCa-2 were purchased from Shanghai Institute of Biochemistry and Cell Biology, Chinese Academy of Sciences (Shanghai, China). PANC‑1 cells were cultured in Dulbecco's modified Eagle's medium (DMEM, Invitrogen) containing 10% fetal bovine serum (FBS, Gibco). CFPAC-1 cells were cultured in Iscove's Modified Dulbecco's Medium (IMEM, Invitrogen) containing 10% FBS (Gibco). MIAPaCa-2 cells were cultured in DMEM (Invitrogen) containing 10% FBS (Gibco), 2.5% Horse Serum (Gibco), and1% sodium pyruvate (Gibco). The normal human pancreatic cell line, HPDE6-C7, was purchased from Genio Biotechnology Co., Ltd. (Guangzhou, China). HPDE6-C7 cells were cultured in minimum essential medium (MEM, Invitrogen) supplemented with 10% FBS (Gibco). The cells were maintained in a humidified incubator at 37˚C in an atmosphere of 5% CO_2_.

### RNA extraction and quantitative real-time PCR

Total RNA was isolated from tissue specimens and cells using the Trizol Reagent (Invitrogen) according to the manufacturer's instructions. RNA was reverse-transcribed into cDNA using the High Capacity cDNA Synthesis kit (Applied Biosystems, Foster City, CA, USA). RT-qPCR was determined using the SYBR Premix Ex Taq kit (Takara Biotechnology Co., Ltd.) and a 7500 Fast Real‑time PCR system (Thermo Fisher Scientific Inc., UK). PCR primer sequences were designed and synthesized by GenScript (Nanjing) Co., Ltd. (Nanjing, China). The primer sequences for qRT-PCR are as follows: Human miR-573 forward, 5′-ACA CTC CAG CTG GGC TGA AGT GAT GTG TAA-3′ and reverse, 5′-TGG TGT CGT GGA GTCG-3′; U6 forward, 5′- CCC CTG GAT CTT ATC AGG CTC-3′ and reverse, 5′- GCC ATC TCC CCG GAC AAAG-3′; Human E2F3 forward, 5'‑ATC GTG CTT CCA TTC CCAG‑3' and reverse, 5'‑CTC TCC ATC TGA CCC CAT CC‑3'; Human GAPDH forward, 5'‑GAA GGT GAA GGT CGG AGTC‑3' and reverse, 5'‑GAA GAT GGT GAT GGG ATT TC‑3'. U6 and GAPDH were used as endogenous controls for miR-573 and E2F3, respectively. The relative expression levels were calculated as a fold change using the 2-ΔΔCt method.

### Cell transfection

MiR‐573 mimics (sense: 5'‐CUGAAGUGAUGUGUAACUGAUCAG‐3', antisense: 5'-CUGAUCAGUUACACAUCACUUCAG-3'), mimic negative control (mimic NC, sense: 5'‐UUUGUACUACACAAAAGUACUG‐3', antisense: 5'-CAGUACUUUUGUGUAGUACAAA-3'), small interfering RNA (siRNA) targeting E2F3 (5'‐GCGATCTCTTCGAGCTTA‐3'), siRNA‐NC (5'‐GGCUCUAGAAAA GCCUAUGC‐3'), and pcDNA3.1-E2F3 were designed and synthesized by RiboBio (Guangzhou, China). PANC‑1, CFPAC-1, and MIAPaCa-2 cells were seeded in 6-well plates at a density of 5×10^5^ cells/well. Lipofectamine 2000 (Invitrogen) was used to transfect these synthetic miRNAs or siRNA into cells. After 48 h of transfection, cells were harvested for subsequent experiments.

### Cell proliferation assay

Cell proliferation was determined using the Cell Counting Kit-8 (CCK-8, Sigma) assay and 5-ethynyl-2'-deoxyuridine (Edu) proliferation assay. Cells were seeded into 96-well plates at a concentration of 5000 cells/well. A volume of 10 μL CCK-8 solution was added to each well and incubated for 3 h at room temperature after culture for 0, 24, 48, 72, and 96 h. The absorbance at 450 nm was measured using a microplate reader (Molecular Devices, Menlo Park, CA, USA). Each experiment was performed three times and the results were averaged. For the Edu assay, cells were seeded into 24-well plates. Edu staining was conducted according to the instructions in the kit (RiboBio). The images were acquired using an inverted fluorescence microscope.

### Colony-forming assay

Cells were seeded into 6-well plates at a density of 1000 and 750 cells/well for PANC‑1 and MIAPaCa-2 cells, respectively, and incubated at 37 °C in a 5% CO_2_ atmosphere in humidified air. PANC‑1 and MIAPaCa-2 cells were cultured for 12 and 9 d, respectively. The culture medium was replaced every 2 d. After 10 d of incubation, the colonies were fixed using 4% paraformaldehyde for 10 min at room temperature and stained with 1% crystal violet for 1 min at room temperature, and the number of colonies in each well was counted.

### Wound-healing assay

Cells were seeded into 6-well plates at a density of 1×10^5^ cells/well and incubated at 37 °C in 5% CO_2_ in humidified air. When the cells reached 80% confluency, the cells were scraped using a 1 mL sterile pipette tip to generate a wound, and washed twice with phosphate-buffered saline. Cells were cultured in a serum-free medium and photographed at 0 and 24 h following generation of the wound using an inverted phase microscope.

### Transwell migration and invasion assay

Transwell migration and invasion assays were performed using the transwell chamber (24-well insert; 8-μm pores; Corning Costar). Matrigel (BD Bioscience, USA) was coated (for invasion assay) or not coated (for migration assay) in the upper layer for 1 h. Around 1 × 10^5^ cells in a total volume of 100 µL FBS-free medium were plated in the top chamber and a medium containing 10% FBS was added to the lower chamber. After incubation for 24 h, cells that migrated/invaded through the membrane were stained using crystal violet, while those that had not migrated or crossed the membrane were removed using cotton swabs. Cells were counted in three different fields of view using an Olympus microscope.

### Bioinformatic analysis and luciferase reporter assay

To predict the potential targets of miR-573, we used four online prediction programs, namely, TargetScan, miRTarBase, miRDB, and miRWalk. E2F3 was predicted as a target of miR-573. Sequences containing wild‐type or a mutant miR‐573‐binding site in the 3'‐UTR of E2F3 were cloned into the pmiR-RB-Report^TM^ vector (Ribobio). Subsequently, cells were co-transfected with miR-573 mimic/negative control and wild-type/ mutant E2F3 using Lipofectamine 2000 reagent (Invitrogen). After 48 h, the activities of Renilla luciferase and firefly luciferase were determined using a dual-luciferase reporter assay system (Promega). Renilla luciferase was used for normalization. All experiments were performed 3 times independently. The aberrantly expressed signaling pathways were confirmed using the Kyoto Encyclopedia of Genes and Genomes (KEGG) through Gene Set Enrichment Analysis (GSEA).

### Differential expression and prognostic analysis

The limma and heatmap package of R was used to screen the different miRNAs between pancreatic cancer tissues and normal pancreas. The Kaplan‑Meier plotter website was used to construct Kaplan‑Meier survival curves of E2F3 expression in patients with pancreatic cancer. The correlation between E2F3 expression and tumor stage in patients with pancreatic cancer was analyzed using the GEPIA online website. The GEPIA boxplot was used to determine the expression of E2F3 in pancreatic cancer tissues as well as adjacent tissues.

### Xenograft model experiment

Six-week-old BALB/c nude mice were acquired from the Laboratory Animal Center of Nantong University and maintained under specific pathogen-free (SPF) conditions. The mice were randomly divided into the miR-573 mimic and mimic NC groups (n = 4 each). A total of 1×10^6^ cells treated with miR-573 mimic or mimic NC were injected subcutaneously into the nude mice. At 30 d post-injection, mice were sacrificed. The tumor volume was evaluated using the following formula: Tumor volume = length×width^2^×0.5. MiR-573 and E2F3 expression of tumors was analyzed using qRT-PCR. All animal studies were conducted in accordance with the National Institutes of Health animal use guidelines, and all animal protocols were approved by the Nantong University Animal Care Committee.

### Statistical analysis

For statistical analysis, Statistical Product and Service Solutions (SPSS 22.0 Software) (SPSS Inc., Chicago, IL, USA) was used. The results are expressed as mean ± standard deviation from at least three independent experiments. Differences were determined using two-tailed Student's *t*-test or one-way ANOVA. P-values < 0.05 were considered significant (*P < 0.05, **P < 0.01, ***P<0.001).

## Results

### MiR-573 expression was downregulated in pancreatic cancer tissues and cell lines

**Figure 1:**
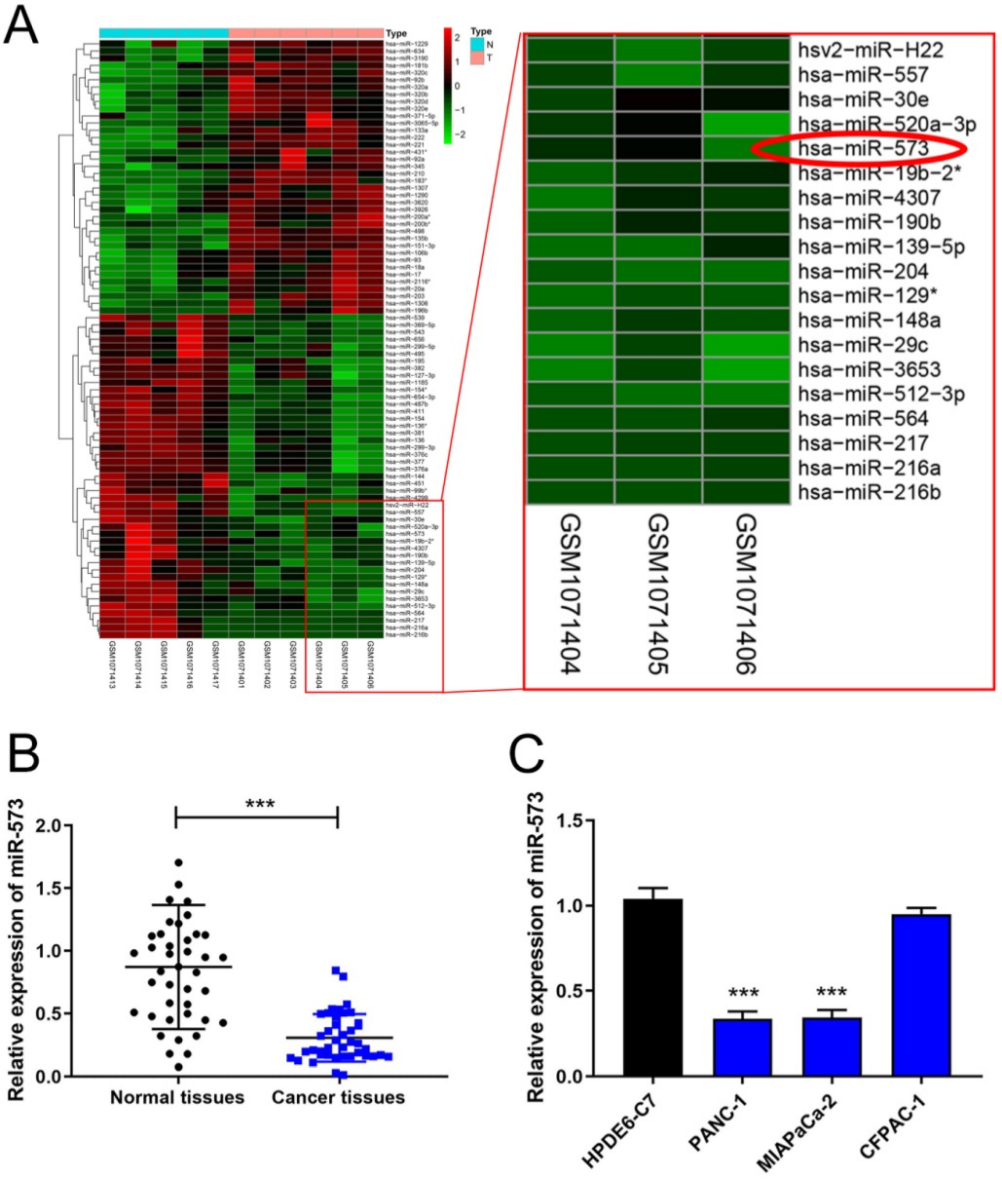
MiR-573 expression is downregulated in pancreatic cancer tissues and cell lines. (A) The heatmap of the miRNA array dataset, GES43796, shows that miR-573 expression is lower in pancreatic cancer than in normal pancreas. (B) MiR-573 expression is significantly downregulated in pancreatic cancer tissues compared to that in matched adjacent normal tissues. (C) MiR-573 expression is downregulated in two pancreatic cancer cell lines (PANC‑1, MIAPaCa-2) compared to that in HPDE6-C7; however, its expression is not significantly downregulated in CFPAC-1.

The miRNA array dataset, GES43796, contains samples of 5 normal pancreas and 6 samples of pancreatic cancer. The heatmaps of different miRNAs are shown in Figure 1A. MiR-573 expression was lower in pancreatic cancer than in normal pancreas. To testify the expression of miR-573 in pancreatic cancer tissues and cell lines, we used qRT-PCR to determine its expression in 41 paired pancreatic cancer tissues and corresponding adjacent normal tissues from patients with pancreatic cancer. The results showed that miR-573 was significantly downregulated in pancreatic cancer tissues compared to the matched adjacent normal tissues (Figure 1B). In addition, based on the median value of expression, miR-573 was divided into corresponding high and low expression groups. By analyzing the relationship between miR-573 and the pathological data of patients, it was found that miR-573 expression was associated with tumor-node-metastasis (TNM) stage, tumor size, and lymph node metastasis (Table 1). Besides, we also checked miR-573 expression in three pancreatic cancer cell lines (PANC‑1, MIAPaCa-2, and CFPAC-1) and a normal human pancreatic ductal epithelial cell line (HPDE6-C7). The results showed that miR-573 expression was significantly dramatically downregulated in PANC‑1 and MIAPaCa-2 compared to that in HPDE6-C7 (Figure 1C). However, its expression was not significantly downregulated in CFPAC-1.

### MiR-573 inhibited the proliferation, migration, and invasion capacities of pancreatic cancer cell lines *in vitro*

**Figure 2:**
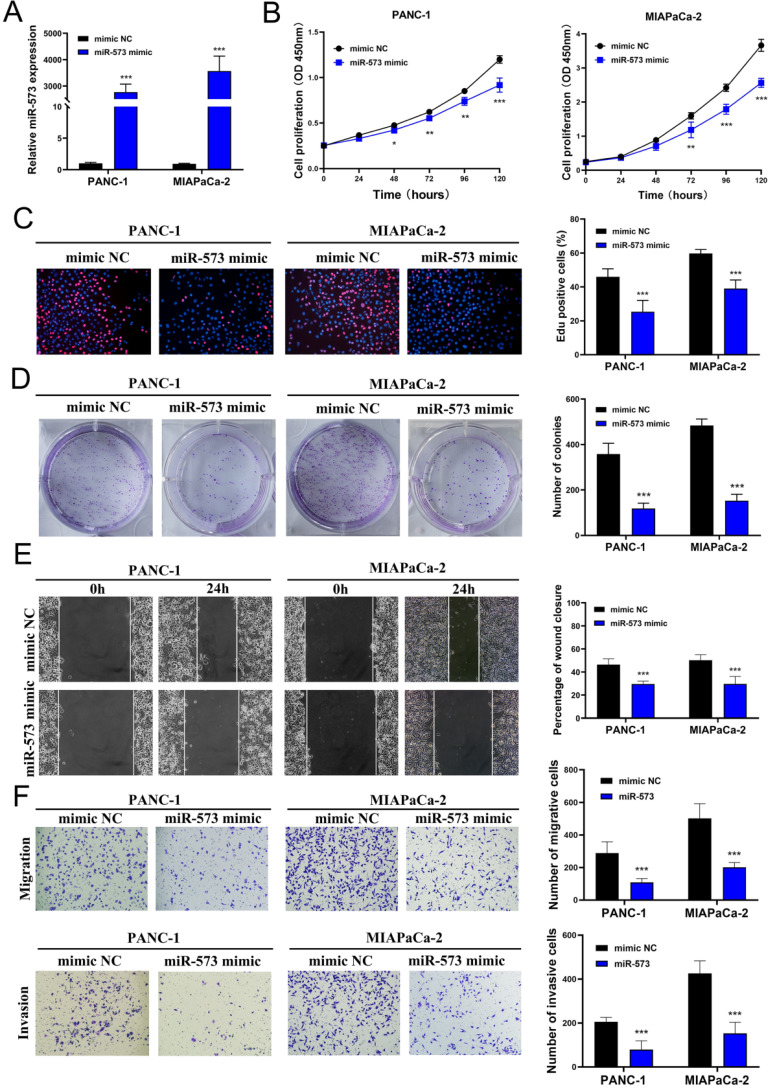
MiR-573 inhibits the proliferation, migration, and invasion capacity of pancreatic cancer cell lines *in vitro*. (A) qRT-PCR results confirm that transfection of miR-573 mimic can significantly increase the expression of miR-573 in pancreatic cancer cell lines. (B) CCK-8 assays show that compared to the mimic NC group, the proliferation ability of PANC-1 and MIAPaCa-2 is remarkably inhibited in miR‑573 mimic group at 72, 96 and 120 h. (C) The number of EdU-positive cells is decreased in the miR‑573 mimic group. (D) MiR‑573 mimic significantly attenuates the colony-forming capacity. (E) Would-healing assay demonstrates that miR-573-overexpressing cells have stronger migratory ability compared to that of the mimic NC group. (F) Transwell migration and invasion assays show that overexpression of miR-573 remarkably suppresses the migration and invasion of PANC-1 and MIAPaCa-2 cells.

To investigate the function of miR-573 in pancreatic cancer cell lines, its overexpressed mimics were designed. PANC-1 and MIAPaCa-2 cell lines were selected to verify the biological function of miR-573 owing to the low miR-573 levels in these cells determined using RT-PCR. MiR‑573 mimics and mimic NC were transfected into two pancreatic cancer cell lines. qRT-PCR results confirmed that transfection of the miR-573 mimic could significantly increase the expression of miR-573 in pancreatic cancer cell lines (Figure 2A). CCK-8 assays showed that compared to the mimic NC group, the proliferation ability of PANC-1 cells was remarkably inhibited in the miR‑573 mimic group at 72, 96, and 120 h (Figure 2B). Similar results were observed in MIAPaCa-2 cells (Figure 2B). The number of Edu-positive cells was decreased in the miR‑573 mimic group (Figure 2C). Besides, the miR‑573 mimic significantly attenuated the colony formation capacity (Figure 2D). In addition, the wound-healing assay demonstrated that the miR-573 overexpressing cells had stronger migratory ability compared to the mimic NC group (Figure 2E). Transwell migration and invasion assays showed that overexpression of miR-573 remarkably suppressed the migration and invasion of PANC-1 and MIAPaCa-2 cells (Figure 2F). These results confirmed that miR-573 inhibited the proliferation, migration, and invasion capacity of pancreatic cancer cells *in vitro*. These findings also suggested that miR-573 might suppress tumor development in the growth and invasion of pancreatic cancer.

### E2F3 is a target gene of miR‑573

**Figure 3:**
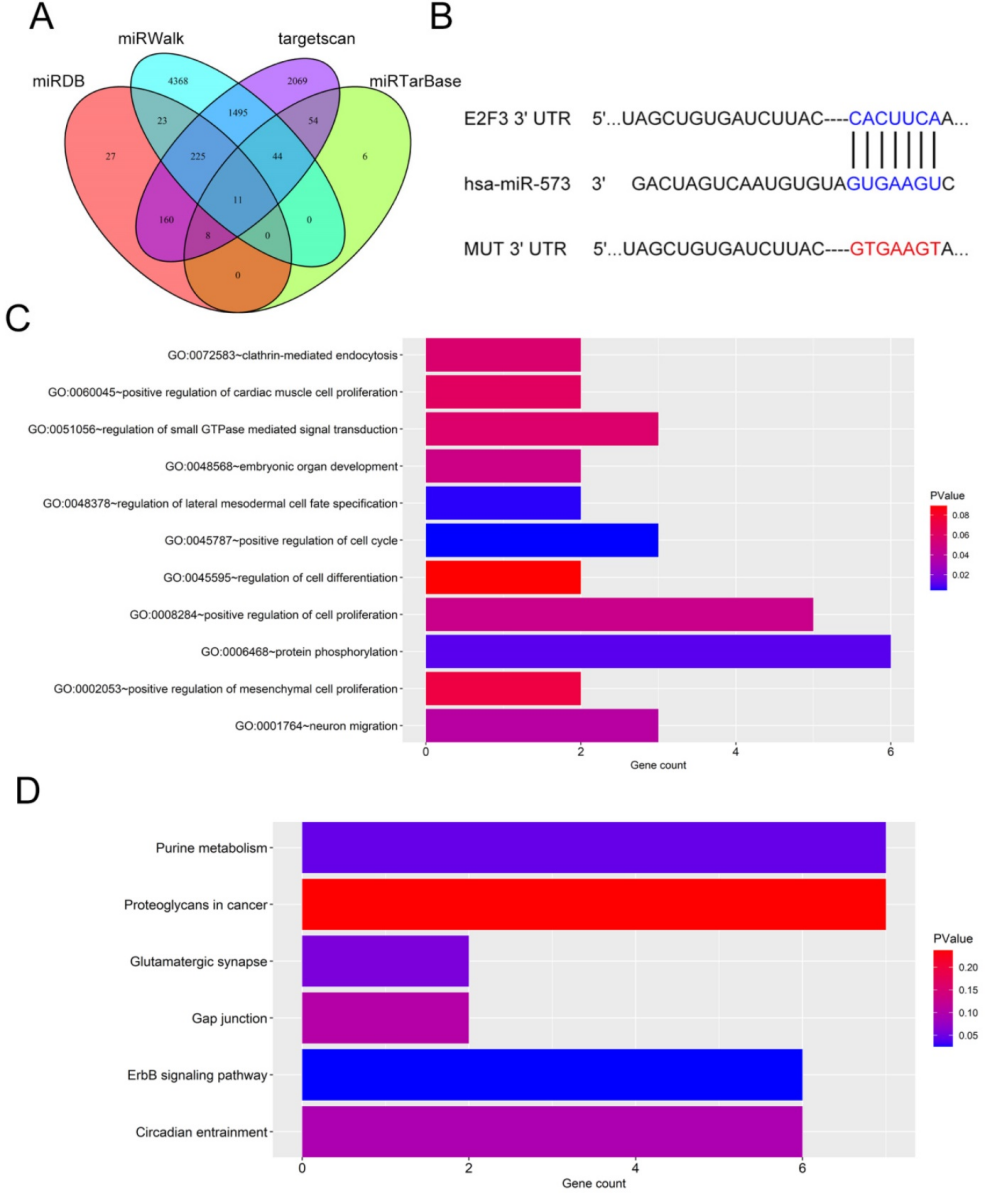
Target genes of miR‑573. (A) We searched for candidate target genes of miR-573 using four different databases and identified that E2F3 is potentially targeted by miR-573. (B) Complementary miR-573 sequence is present in the 3′-UTR of E2F3 mRNA. (C) GO enrichment analysis of target genes. (D) KEGG enrichment analysis of target genes.

**Figure 4:**
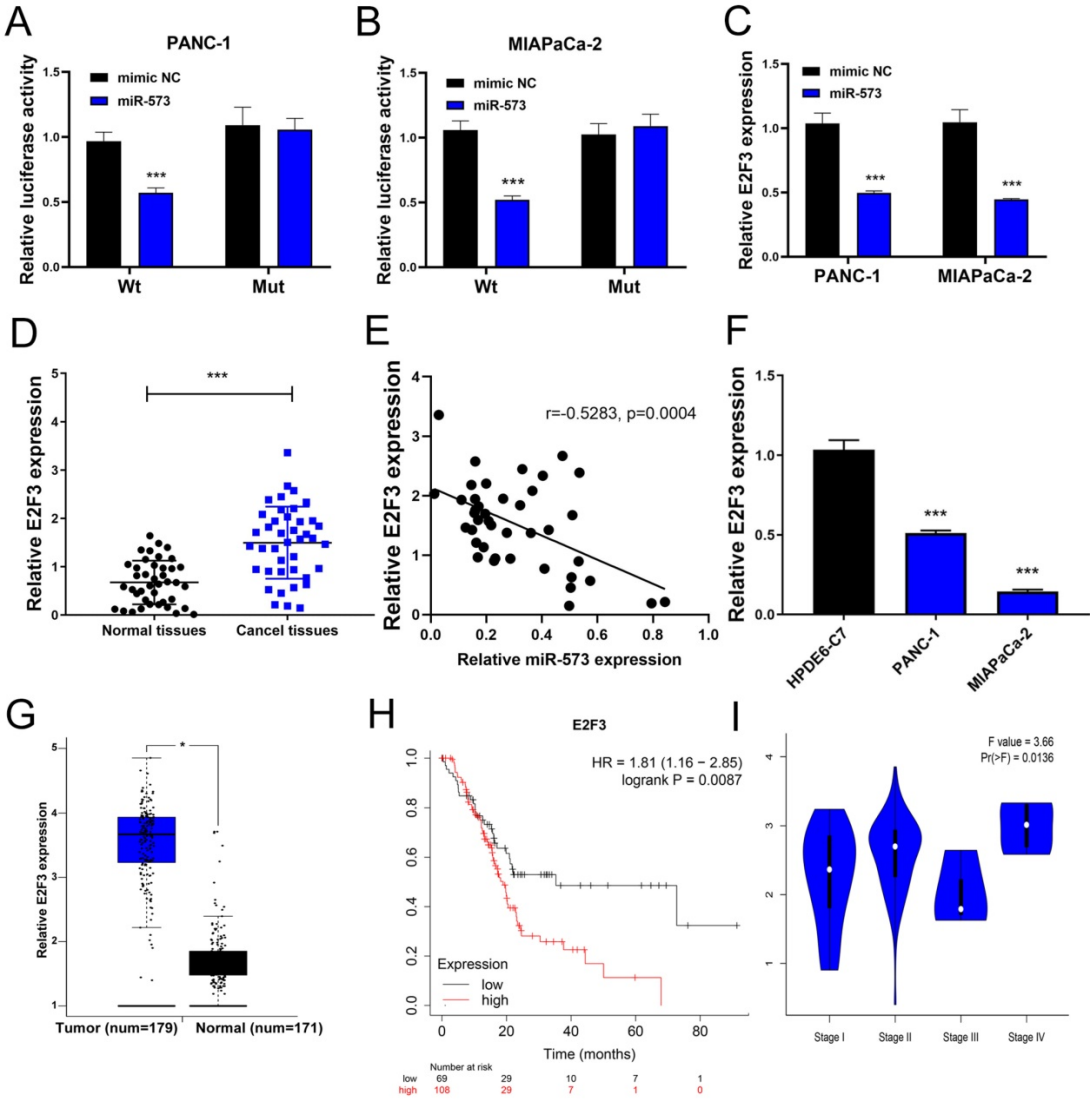
E2F3, a target gene of miR-573, is upregulated in human pancreatic cancer tissues and cell lines, and prognostic values of E2F3 in pancreatic cancer. (A, B) Luciferase reporter assay shows that the overexpression of miR-573 significantly reduces luciferase activity in PANC-1 and MIAPaCa-2 cells treated with pmiR-RB-E2F3-3′ UT Wt, but has no effect on that of pmiR-RB-E2F3-3′ UT Mut. (C) Overexpression of miR-573 significantly downregulates E2F3 expression in PANC-1 and MIAPaCa-2 cells. (D) qRT-PCR shows that expression level of E3F3 in pancreatic cancer tissues is higher than that in normal tissues. (E) Pearson's correlation analysis shows a significant negative correlation between the expression of E2F3 and miR-573 in pancreatic cancer tissues (Figure 4E). (F) E2F3 expression is considerably upregulated in PANC‑1 and MIAPaCa-2 compared to HPDE6-C7 cells. (G) MRNA expression levels of E2F3 are significantly higher in patients with pancreatic cancer based on the analysis of 171 normal pancreatic tissues and 179 pancreatic cancer tissues from the GEPIA online website. (H) Kaplan-Meier analysis shows that E2F3 displays a significant correlation with the overall survival of patients with pancreatic cancer. Patients with a high level of E2F3 suffer worse prognosis than those with low levels. (I) The expression level of E2F3 displays a significant correlation with the tumor stage in patients with pancreatic cancer.

Next, the downstream mechanism of miR-573 in pancreatic cancer was investigated. We searched the candidate target genes of miR-573 using four different databases. We identified that E2F3 was potentially targeted by miR-573 (Figure 3A). We found that a complementary miR-573 sequence was present in the 3′-UTR of E2F3 mRNA (Figure 3B). GO enrichment analysis indicated that the target genes were enriched in the regulation of cell cycle, cell proliferation, and cell-differentiation related GO terms (Figure 3C). KEGG enrichment analysis suggested that these genes were involved in the ErbB signaling pathway, purine metabolism, glutamatergic synapse, circadian entrainment, gap junction, and proteoglycans in cancer (Figure 3D). To further confirm the relationship between E2F3 and miR-573, a luciferase reporter assay was performed. The results showed that the over-expression of miR-573 significantly reduced the luciferase activity in PANC-1 and MIAPaCa-2 cells treated with pmiR-RB-E2F3-3′ UT Wt (Figures 4A and B), but had no effect on that of pmiR-RB-E2F3-3′ UT Mut. In addition, we performed qRT-PCR to quantify the expression levels of E3F3 mRNA in PANC-1 and MIAPaCa-2 cells transfected with miR-573 mimics or mimic NC. Results showed that over-expression of miR-573 significantly down-regulated E2F3 expression in PANC-1 and MIAPaCa-2 cells (Figure 4C). Besides, Pearson's correlation analyses showed a significant negative correlation between the expression of E2F3 and miR-573 in pancreatic cancer tissues (Figure 4E, r=-0.5283, p=0.0004). These results collectively indicated that E2F3 is a target gene of miR-573 in pancreatic cancer.

### E2F3 is up-regulated in human pancreatic cancer tissues and cell lines and prognostic values of E2F3 in pancreatic cancer

We further detected the expression of E2F3 in pancreatic cancer tissues and corresponding adjacent normal tissues using qRT-PCR. The results showed that the expression level of E3F3 in pancreatic cancer tissues was higher than that in normal tissues (Figure 4D). Besides, we also determined E2F3 expression in PANC‑1, MIAPaCa-2, and HPDE6-C7 cells. The results showed that E2F3 expression was dramatically upregulated in PANC‑1 and MIAPaCa-2 compared to that in HPDE6-C7 cells (Figure 4F). By analyzing 171 normal pancreatic tissues and 179 pancreatic cancer tissues on the GEPIA website, we found the mRNA expression levels of E2F3 were significantly higher in patients with pancreatic cancer than in the normal control patients (Figure 4G). The Kaplan-Meier analysis showed that E2F3 displayed a significant correlation with the overall survival of patients with pancreatic cancer. Patients with a high level of E2F3 suffered worse prognosis than those without (Figure 4H, HR=1.81, p=0.0087). Besides, we analyzed the correlation between E2F3 expression and tumor stage in patients with pancreatic cancer on the GEPIA website. The results demonstrated that the expression level of E2F3 displayed a significant correlation with the tumor stage in patients with pancreatic cancer (Figure 4I). These results collectively demonstrated that E2F3 levels are up-regulated in pancreatic cancer.

### Effects of E2F3 on the proliferation, migration, and invasion capacity of pancreatic cancer cell lines and the proliferation rescue experiment

**Figure 5:**
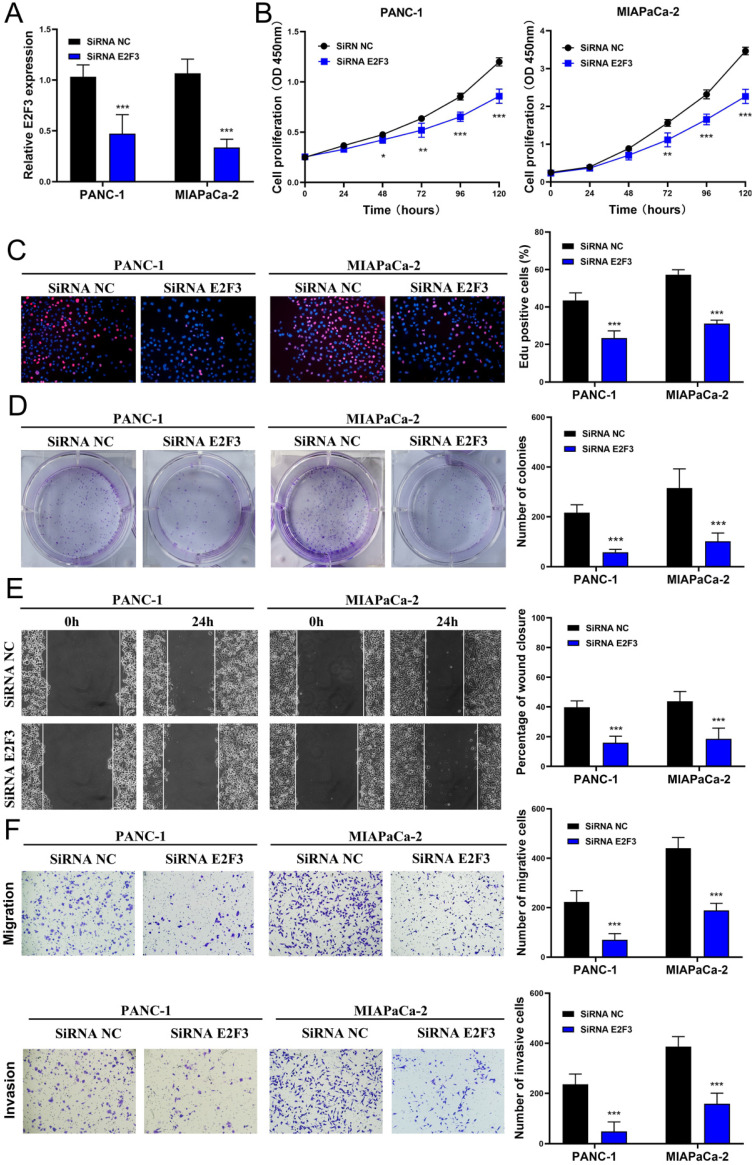
Downregulation of E2F3 inhibits proliferation, migration, and invasion capacity of pancreatic cancer cell lines *in vitro*. (A) E2F3 expression is downregulated in pancreatic cancer cells after transfection with E2F3 siRNA. (B) CCK-8 assay shows that E2F3 siRNA inhibits pancreatic cancer cell growth. (C) The number of Edu-positive cells is reduced in cells transfected with E2F3 siRNA. (D) E2F3 inhibition significantly attenuates the colony-forming capacity. (E) Would-healing assay demonstrates that E2F3 inhibition decreases migratory ability. (F) The number of migrated and invasive cells decreases after transfection with E2F3 siRNA.

**Figure 6:**
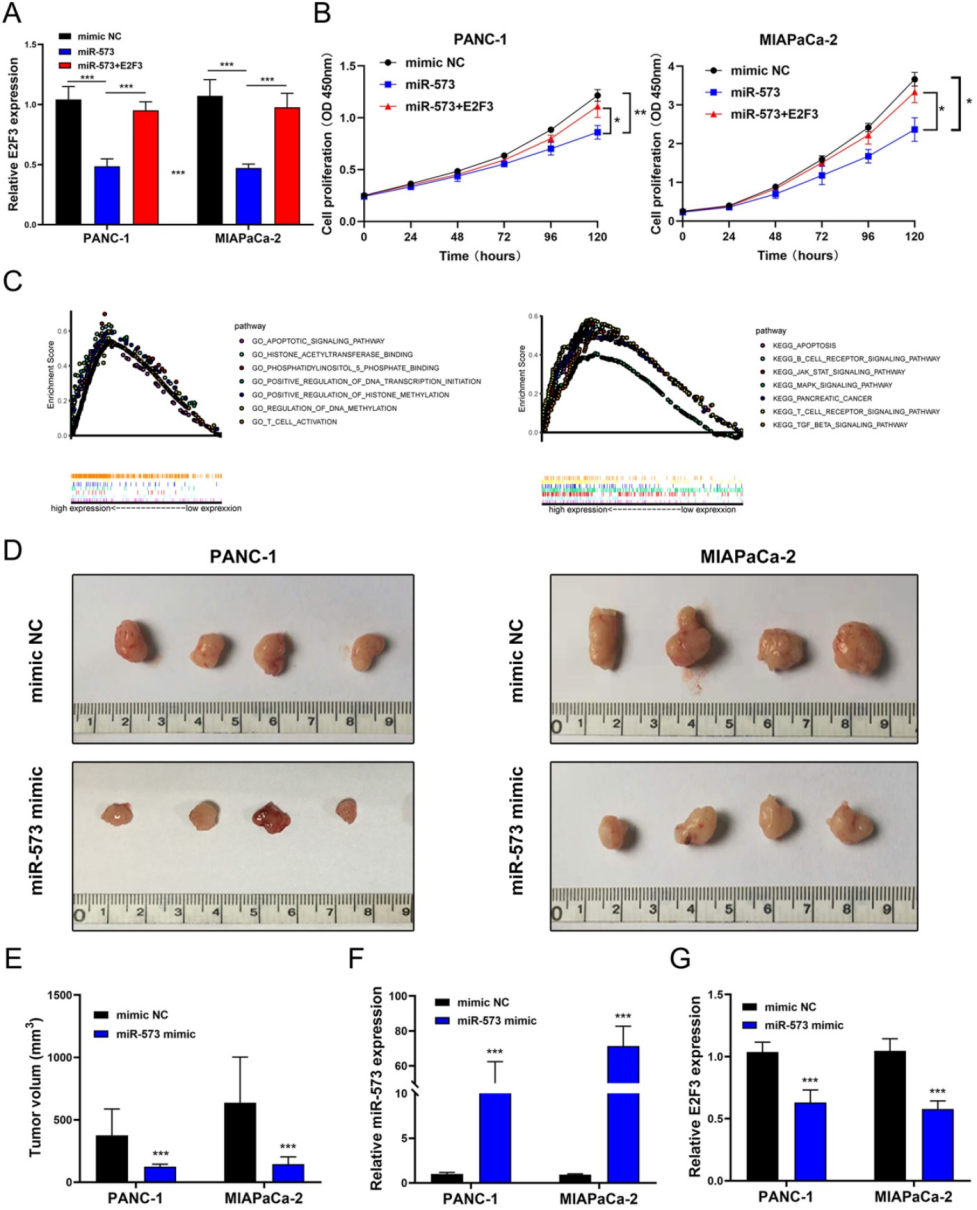
(A) qRT-PCR shows that overexpression of E2F3 in miR-573 mimic-transfected cells restores E2F3 expression. (B) Overexpression of E2F3 attenuates the effect of the miR-573 mimic on the cell proliferation ability of PANC-1 and MIAPaCa-2 cells. (C) GSEA analysis of E2F3 in GO analyses and KEGG analyses. (D, E) Mice injected with miR-573 overexpressed pancreatic cancer cells show decreased tumor volume compared to the negative control. (F) MiR-573 expression is significantly increased in the miR-573 upregulating group compared to the negative group. (G) E2F3 expression is evidently reduced in miR-573 upregulating group compared to the negative group.

To explore whether E2F3 is a key regulator of cellular behavior in pancreatic cancer cells, E2F3 siRNA was used. We observed a significantly down-regulated expression of E2F3 in pancreatic cancer cells that were transfected with E2F3 siRNA (Figure 5A). The CCK-8 assay showed that E2F3 siRNA substantially inhibited pancreatic cancer cell growth (Figure 5B). The number of Edu-positive cells was reduced in cells transfected with E2F3 siRNA (Figure 5C). Besides, E2F3 inhibition significantly attenuated the colony formation capacity (Figure 5D). In addition, the wound-healing assay demonstrated that E2F3 inhibition decreased the migratory ability (Figure 5D). Moreover, the number of migrated and invasive cells was decreased after transfection with E2F3 siRNA (Figure 5F). These results confirmed that E2F3 inhibition suppressed the proliferation, migration, and invasion capacity of pancreatic cancer cells. To evaluate the effect of E2F3 on miR-573 over-expression, pancreatic cancer cells were co-transfected with an miR-573 mimic or miR-NC and pcDNA3.1-E2F3. QRT-PCR showed that over-expression of E2F3 in miR-573 mimic transfected cells restored E2F3 expression (Figure 6A). In addition, we found that over-expression of E2F3 attenuated the effect of the miR-573 mimic on the cell proliferation ability of PANC-1 and MIAPaCa-2 cells (Figure 6B). Besides, GSEA analysis (Figure 6C) indicated that the primary increased functions of E2F3 in GO analyses were related to the apoptotic signaling pathway, histone acetyltransferase binding, phosphatidylinositol phosphate binding, regulation of DNA transcription initiation, regulation of histone methylation, regulation of DNA methylation, and T cell activation. The primary increased functions of E2F3 in KEGG analyses were related to apoptosis, B cell receptor signaling pathway, JAK-STAT signaling pathway, MAPK signaling pathway, pancreatic cancer, T cell receptor signaling pathway, and TGF beta signaling pathway.

### MiR-573 inhibited xenograft tumor formation *in vivo*

To further investigate the role of miR-573 in the proliferation process of pancreatic cancer cells *in vivo*, PANC‑1 and MIAPaCa-2 cell lines stably overexpressing miR-573 were inoculated subcutaneously into the flanks of nude mice to generate tumor ectopically. The mice were found to have decreased tumor volume compared to those in the negative control group (Figures 6D and E). MiR-573 expression was significantly increased in the miR-573 up-regulating group compared to that in the negative group (Figure 6F). E2F3 expression was evidently reduced in the miR-573 up-regulating group compared to that in the negative group (Figure 6G). These findings suggested that miR-573 inhibited tumor formation *in vivo*.

## Discussion

Pancreatic cancer is one of the most serious malignant tumors among digestive-system tumors. However, the molecular mechanisms underlying the carcinogenesis and development of pancreatic cancer have not yet been elucidated. Evidence has shown that non-coding RNAs including miRNAs, lncRNAs, and circRNAs are involved in the development of cancers 16. Numerous miRNAs have been shown to play key roles in cancer development by targeting tumor suppressor genes or oncogenes 17. In pancreatic cancer, the roles of a series of miRNAs have been investigated. For instance, miR-18a induces epithelial-mesenchymal transition like cancer stem cell phenotype via regulating the RKIP pathway 18. miR-216a mediates the upregulation of TSPAN1 via transcriptional regulation of ITGA2 19, and miR-193a-5p promotes cell metastasis through SRSF6-mediated alternative splicing of OGDHL and ECM1 20. Previous studies have demonstrated that miR-573 is aberrantly expressed and plays significant roles in different cancers 7, 8, 21. Nevertheless, its roles in pancreatic cancer are still under investigation. In the current study, miR-573 expression was significantly decreased in pancreatic cancer tissues and cell lines compared to that in paired normal adjacent tissues and HPDE6-C7 cells. Further studies revealed that over-expression of miR-573 inhibited the proliferation, migration, and invasion of pancreatic cells *in vitro* and tumorigenicity *in vivo*. These results, for the first time, suggested that miR-573 might be a potential tumor suppressor in pancreatic cancer.

To elucidate the molecular mechanisms underlying the involvement of miR-573 in pancreatic cancer, we used bioinformatics analysis to predict that E2F3 is a putative target gene for miR-573. E2F3 is a transcription factor that can regulate DNA replication, mitosis, apoptosis, and centrosome duplication. Overexpression of E2F3 is frequently observed in various cancers. For instance, miR‑34a expression affects breast cancer invasion *in vitro* and patient survival via the down-regulation of E2F3 expression 22. QKI-6 inhibits the malignant behavior of bladder cancer cells through down-regulating E2F3 and NF-κB signaling 23. The high expression of E2F3 in pancreatic cancer samples was determined from the GEPIA website. Our results confirmed that E2F3 expression was significantly upregulated in pancreatic cancer tissues and cell lines. Down-regulation of E2F3 inhibited proliferation, migration, and invasion of pancreatic cells. Moreover, a luciferase reporter assay corroborated that miR-573 directly targeted E2F3 and inhibited its expression. Overall, the present study reveals that E2F3 is directly and negatively regulated by miR-573. To the best of our knowledge, this is the first study to demonstrate the potential link between miR-573 and E2F3 in pancreatic cancer.

## Conclusions

In conclusion, this study illustrated that miR-573 functions as a tumor suppressor and can negatively regulate E2F3 to inhibit cell proliferation and invasion capacity in pancreatic cancer. The miR-573/E2F3 axis might serve as a novel biomarker to predict the progression and prognosis of pancreatic cancer, as well as a potent therapeutic target in the treatment of pancreatic cancer.

## Figures and Tables

**Table 1:** Correlation of the expression of miR-573 with clinicopathological parameters

Characteristics	Number	MiR-573 expression		P value
		Low	High	
**Gender**				
Female	16	10	6	0.509
Male	25	13	12	
**Age (years)**				0.326
<60	14	9	5	
≥60	27	13	14	
**TNM stage**				0.019*
I-II	20	7	13	
III-IV	21	15	6	
**Tumor size (cm)**				0.017*
≤4	22	8	14	
>4	19	14	5	
**Lymph node metastasis**				0.042*
No	13	10	3	
Yes	28	12	16	
**Differentiation**				
High	11	4	7	0.086
Moderate	14	6	8	
Low	16	12	4	

## References

[1] Kamisawa T, Wood LD, Itoi T, Takaori K (2016). Pancreatic cancer. Lancet.

[2] Siegel RL, Miller KD, Jemal A (2019). Cancer statistics, 2019. CA: a cancer journal for clinicians.

[3] Huang W (2017). MicroRNAs: Biomarkers, Diagnostics, and Therapeutics. Methods in molecular biology.

[4] Jonas S, Izaurralde E (2015). Towards a molecular understanding of microRNA-mediated gene silencing. Nature reviews Genetics.

[5] Gong R, Jiang Y (2020). Non-coding RNAs in Pancreatic Ductal Adenocarcinoma. Frontiers in oncology.

[6] Wang R, Wen B, Sun D (2019). miR-573 regulates cell proliferation and apoptosis by targeting Bax in nucleus pulposus cells. Cellular & molecular biology letters.

[7] Gao X, Zhao S, Yang X, Zang S, Yuan X (2018). Long non-coding RNA FLVCR1-AS1 contributes to the proliferation and invasion of lung cancer by sponging miR-573 to upregulate the expression of E2F transcription factor 3. Biochemical and biophysical research communications.

[8] Chen P, Wang R, Yue Q, Hao M (2018). Long non-coding RNA TTN-AS1 promotes cell growth and metastasis in cervical cancer via miR-573/E2F3. Biochemical and biophysical research communications.

[9] Wang L, Song G, Tan W, Qi M, Zhang L, Chan J (2015). MiR-573 inhibits prostate cancer metastasis by regulating epithelial-mesenchymal transition. Oncotarget.

[10] Hu YW, Chen ZP, Hu XM, Zhao JY, Huang JL, Ma X (2015). The miR-573/apoM/Bcl2A1-dependent signal transduction pathway is essential for hepatocyte apoptosis and hepatocarcinogenesis. Apoptosis: an international journal on programmed cell death.

[11] Tammali R, Saxena A, Srivastava SK, Ramana KV (2010). Aldose reductase regulates vascular smooth muscle cell proliferation by modulating G1/S phase transition of cell cycle. Endocrinology.

[12] Liu J, Si L, Tian H (2018). MicroRNA-148a inhibits cell proliferation and cell cycle progression in lung adenocarcinoma via directly targeting transcription factor E2F3. Experimental and therapeutic medicine.

[13] Jin Y, Wei J, Xu S, Guan F, Yin L, Zhu H (2019). miR2103p regulates cell growth and affects cisplatin sensitivity in human ovarian cancer cells via targeting E2F3. Molecular medicine reports.

[14] Guo J, Zhang J, Yang T, Zhang W, Liu M (2020). MiR-22 suppresses the growth and metastasis of bladder cancer cells by targeting E2F3. International journal of clinical and experimental pathology.

[15] Shi L, Zhu H, Shen Y, Dou X, Guo H, Wang P (2020). Regulation of E2F Transcription Factor 3 by microRNA-152 Modulates Gastric Cancer Invasion and Metastasis. Cancer management and research.

[16] Anastasiadou E, Jacob LS, Slack FJ (2018). Non-coding RNA networks in cancer. Nature reviews Cancer.

[17] Bhaskaran M, Mohan M (2014). MicroRNAs: history, biogenesis, and their evolving role in animal development and disease. Veterinary pathology.

[18] Kang H, Ma D, Zhang J, Zhao J, Yang M (2020). MicroRNA-18a induces epithelial-mesenchymal transition like cancer stem cell phenotype via regulating RKIP pathway in pancreatic cancer. Annals of translational medicine.

[19] Wang S, Liu X, Khan AA, Li H, Tahir M, Yan X (2020). miR-216a-mediated upregulation of TSPAN1 contributes to pancreatic cancer progression via transcriptional regulation of ITGA2. American journal of cancer research.

[20] Li M, Wu P, Yang Z, Deng S, Ni L, Zhang Y (2020). miR-193a-5p promotes pancreatic cancer cell metastasis through SRSF6-mediated alternative splicing of OGDHL and ECM1. American journal of cancer research.

[21] Lu Z, Luo T, Nie M, Pang T, Zhang X, Shen X (2015). TSPAN1 functions as an oncogene in gastric cancer and is downregulated by miR-573. FEBS letters.

[22] Han R, Zhao J, Lu L (2020). MicroRNA34a expression affects breast cancer invasion *in vitro* and patient survival via downregulation of E2F1 and E2F3 expression. Oncology reports.

[23] Shi F, Deng Z, Zhou Z, Jiang CY, Zhao RZ, Sun F (2019). QKI-6 inhibits bladder cancer malignant behaviours through down-regulating E2F3 and NF-kappaB signalling. Journal of cellular and molecular medicine.

